# In Situ Measurements of Strain Evolution in Graphene/Boron Nitride Heterostructures Using a Non-Destructive Raman Spectroscopy Approach

**DOI:** 10.3390/nano12173060

**Published:** 2022-09-03

**Authors:** Marc Mezzacappa, Dheyaa Alameri, Brian Thomas, Yoosuk Kim, Chi-Hou Lei, Irma Kuljanishvili

**Affiliations:** 1 Department of Aerospace and Mechanical Engineering, Saint Louis University, St. Louis, MO 63103, USA; 2 Department of Physics, Saint Louis University, St. Louis, MO 63103, USA; 3 Department of Physics, College of Science, University of Misan, Maysan 62001, Iraq

**Keywords:** 2D heterostructures, CVD, in situ Raman spectroscopy, strain evolution, non-destructive mechanical testing

## Abstract

The mechanical properties of engineered van der Waals (vdW) 2D materials and heterostructures are critically important for their implementation into practical applications. Using a non-destructive Raman spectroscopy approach, this study investigates the strain evolution of single-layer graphene (SLGr) and few-layered boron nitride/graphene (FLBN/SLGr) heterostructures. The prepared 2D materials are synthesized via chemical vapor deposition (CVD) method and then transferred onto flexible polyethylene terephthalate (PET) substrates for subsequent strain measurements. For this study, a custom-built mechanical device-jig is designed and manufactured in-house to be used as an insert for the 3D piezoelectric stage of the Raman system. In situ investigation of the effects of applied strain in graphene detectable via Raman spectral data in characteristic bonds within SLGr and FLBN/SLGr heterostructures is carried out. The in situ strain evolution of the FLBN/SLGr heterostructures is obtained in the range of (0–0.5%) strain. It is found that, under the same strain, SLG exhibits a higher Raman shift in the *2D* band as compared with FLBN/SLGr heterostructures. This research leads to a better understanding of strain dissipation in vertical 2D heterostacks, which could help improve the design and engineering of custom interfaces and, subsequently, control lattice structure and electronic properties. Moreover, this study can provide a new systematic approach for precise in situ strain assessment and measurements of other CVD-grown 2D materials and their heterostructures on a large scale for manufacturing a variety of future micro- and nano-scale devices on flexible substrates.

## 1. Introduction

Two-dimensional (2D) materials, in the form of single-layered material or the building block of hybrid systems, have been explored for energy, catalysis, electronics, and medical applications [1,2,3,4]. Specifically, the superior properties and performance of van der Waals (vdW) materials have continued to motivate researchers and inspired them to make valuable discoveries advantageous for numerous applications in different fields [5,6]. For example, the transparency, different electronic band gaps, lightweight, high Young’s modulus, and high strength are some of the most desirable properties of 2D materials for practical and industrial implementations [5,7,8], specific to 2D nanostructures compared with their bulk counterparts. These properties make graphene (Gr); boron nitride (BN); and other vdW structures, such as transition metal dichalcogenides (TMDCs), promising materials for potential electronic, optoelectronic, and mechanical applications [3,7,9,10,11,12]. Although 2D materials have shown outstanding intrinsic properties, it is essential to understand their potential in terms of durability for long-lasting and reliable applications. Much effort has been made to investigate the effects of external mechanical forces on the possible changes in the band structure, electronic performance, and elastic behaviors of 2D materials, owing to the deformation. Several approaches have been devoted to studying the mechanical properties of 2D materials, such as fracture strength, Young’s modulus, and strain, using experimental and theoretical methods. Experimental techniques include tensile test until failure, atomic force microscopy (AFM)-assisted nanoindentation [8], and Raman spectroscopy-enabled measurements [10,13].

In theoretical studies, for instance, Liu et al. have reported that applying a small bending strain only (about 0.25 nN nm) on Gr sheets can open a small bandgap of ~0.05 eV owing to the reduction in the π orbital spacing (orbital electrons) [14]. Another theoretical study has shown that applying shear strains can induce the bandgap in the single-layer Gr (SLGr) up to 4 eV, and up to 6 eV under the shear and uniaxial strains [15]. Falin et al., on the other hand, has experimentally demonstrated the thickness impact of single- and multi-layer Gr (SLGr, MLGr) and BN thin films. For example, it was reported that multi-layer BN (up to 9 L) can have a strength similar to a single layer, in contrast to Gr, whose strength decreased significantly with the increased number of layers [10]. It has also been experimentally shown that a nonlinear stress–strain behavior of free-standing SLGr has second- and third-order elastic stiffness of (~340 N/m) and (~690 N/m), respectively (measured via ‘nanomechanical indentation’ approach using AFM), and further confirmed that SLGr is the hardest 2D material, with a Young’s modulus of 1.0 TPa [16]. On the other hand, hexagonal boron nitride (h-BN), an ultra-wide bandgap material, has been shown to possess excellent optical sensitivity, remarkable chemical and thermal stability, and outstanding mechanical properties such as strength. BN has atomically flat surfaces comparable to Gr and a relatively similar lattice structure with a mismatch of only ~1.7% or less [9,10,17,18]. Additionally, h-BN can possess piezoelectric property under a significant strain (up to 6.2%) owing to the wide bandgap and asymmetric characteristics [19].

Several theoretical and experimental studies have recently emphasized the importance of investigating the specific physical properties of different 2D materials combined in the heterostructures. For example, studies have utilized h-BN to serve as a dielectric substrate for graphene devices to modify the intrinsic optical, electronic, and mechanical properties of graphene films [11,12,20,21]. Understanding the mechanical properties of 2D heterostructures is critically important as more exotic 2D materials are discovered. Studies involving complex architectures are being designed and implemented for various applications [22,23,24]. While standard methods of determining the mechanical strength of materials often rely on somewhat destructive methods such as fracture, inelastic deformation, or failure, the fundamental physical and mechanical characteristics of individual 2D nanomaterials can be studied using non-destructive spectroscopic techniques. Such non-destructive methods are especially useful for 1D or 2D nanomaterials thanks to their nano-scale nature and the apparent difficulty in manipulating them in a controllable way using conventional tools [25]. Two of the most frequently used methods that have been utilized to evaluate the mechanical properties of 2D materials are AFM [16,26,27] and Raman spectroscopy [28,29,30]. With the AFM method, the AFM-tip is utilized in situ to apply direct force to the suspended 2D materials to extract the Young’s modulus and evaluate the stress–strain behavior and failure parameters. On the other hand, using the Raman spectroscopy technique, the mechanical properties of 2D materials can be studied based on the distinctive changes in the modes/bands of the spectra. The bond length within the material can be affected when strain is applied, resulting in shifts of the vibrational modes/bands of that material to a lower or higher wavenumber/frequency. An interesting work by Neumann et al. studied the nanometer-scale strain variations using a magnetic field and deduced the information about the local strain homogeneity, and the structural quality of Gr was evaluated [28]. The Raman spectroscopy technique can be considered as non-destructive and non-invasive to the samples under investigation. In general, each material has its distinct Raman signature under specific laser excitation, which is related to the molecular vibrations in the sample resulting from the frequency difference between the laser wavelength probing the sample and the detected scattered light. These Raman active vibrational modes are revealed as peaks in the Raman spectral plots. When samples are exposed to external effects such as applied strain, Raman peaks may shift towards lower or higher frequencies. For instance, G and 2D Raman modes/peaks of Gr thin films are shifted when bond lengths or angles between carbon atoms are impacted by applied strain [31,32,33,34]. Besides observing the strain effect, the resonant Raman spectroscopy technique can provide valuable information about the number of layers, band structure, interlayer coupling, and levels of doping or defects in the measured materials [35,36,37]. In addition, the measurements using the Raman spectroscopy technique can be performed much faster and probe larger-sized samples as compared with AFM-enabled methods, where often smaller randomly deposited 2D flakes of materials are made with a “scotch tape” mechanical exfoliation method. Hence, studying larger-sized samples could be more beneficial for specific types of mechanical testing in many different 2D systems, while also providing chemical, structural, and other identifiable characteristics. Moreover, the Raman approach allows for simultaneous probing of each individual material’s characteristics in heterostructure samples. A similar approach to Raman studies of other 2D materials under mechanical strain has also been investigated [38,39].

It is essential to fabricate clean, flat, intact, continuous single- or few-layered heterostructures on flexible substrates to study their mechanical properties. In general, most fabrication methods of vdW materials and their heterostructures fall into two categories: top-down or bottom-up synthesis techniques [25,40]. As we have mentioned earlier, research related to the mechanical characteristics of vdW heterostructures is often performed on samples prepared by the mechanical exfoliation method [33,41,42]. Mechanical exfoliation is one of the most widely used methods for producing 2D materials owing to the simplicity and the highest quality of the flakes. The method, however, is generally low yield; not scalable; and not applicable to the production of large, continuous films with a controlled number of layers. In contrast, the chemical vapor deposition (CVD) method can synthesize good-quality uniform films with a controlled number of layers.

Our study focuses on heterostructures of FLBN/SLGr thin films prepared using the CVD method and then transferred onto flexible PET substrates. Using the Raman spectroscopy technique, the strain evolution effects in the fabricated FLBN/SLGr were investigated. A custom-built mechanical device, the so-called ‘jig’, was designed for this study to be mounted directly onto the 3D-piezoelectric stage of a Raman spectroscopy system. This mechanical ‘jig’ is capable of applying a micron resolution displacement/bending, which is more precise compared with other reported studies [12,34,43]. This, in turn, allowed for a steady and accurate application of strain during the spectral measurements on the loaded thin films within the in situ Raman system.

In the present work, a resonant Raman spectroscopy was employed to study strain evolution in our samples by measuring shifts in the location of the *2D* band/peak with high precision, which subsequently allowed us to determine the amount of strain. The evolution of strain imposed on the sample was monitored as a function of applied ‘stress’, resulting from the strain induced within the device via micrometer-controlled displacement, thus producing the incremental bending of the SLGr and FLBN/SLGr samples on the PET substrate. This shift was determined to be ~17.9 cm^−1^/% strain for SLGr versus approximately ~12.5 cm^−1^/% strain for the FLBN/SLGr heterostructure. The results provide an important insight into the physical and mechanical performances of 2D heterostructures, which could be compared to the stress–strain curves [34,38,39,44]. To the best of our knowledge, this is the first investigation that explores the mechanical characteristics of FLBN/SLGr heterostructures prepared via CVD synthesis and studied with a non-destructive Raman spectroscopy technique.

## 2. Materials and Methods

### 2.1. Graphene and Boron Nitride CVD Synthesis

Graphene (SLGr) films were grown on 250 µm thick copper (Cu) foil substrates using a home-built CVD system. The CVD system is equipped with a three-zone furnace (Thermo Scientific Lindberg/Blue M, Waltham, MA, USA) and a quartz tube (6 ft L, 22 mm ID, 25 mm OD) (Technical Glass Products, Painesville, OH, USA). A digital mass flow controller (Sierra Instrument, Monterey, CA, USA) was used to synthesize SLGr and FLBN thin films. To synthesize an SLGr layer, the CVD growth process was performed under ambient pressure, 950 °C growth temperature, and a mixture of argon/methane (Ar/CH_4_) gases with the ratio of 1800/77 (sccm) and 20 min growth time. After the growth, the CVD temperature was gradually cooled down to room temperature under the protection of Ar flow at 450 sccm.

Similarly, boron nitride (FLBN) films were also separately prepared on a 250 µm thick Cu-foil substrate using the same CVD system. For FLBN film production, ammonia borane powder was used as a solid precursor at the upstream zone of the CVD quartz tube, where the temperature was fixed at around 200 °C. The evaporated precursor was carried out by a mixture of Ar/H_2_ gases with a ratio of 300/50 (sccm) to a higher-temperature zone (990 °C) for a 40 min growth time. After the growth, the source precursor was pulled out of the upstream zone for rapid cooling, while the CVD chamber was cooled naturally under the protection of the Ar gas flow of 500 sccm.

### 2.2. Graphene and Boron Nitride Transfer

The CVD-grown films (SLGr or FLBN) on Cu-foils were transferred into a 125 µm thick (2.5 cm × 1.5 cm rectangular) polyethylene terephthalate (PET) substrate. Prior to the transfer, the PET substrates were sputter-coated (model HUMMER Ⅵ-A, Anatech LTD, Alexandria, VA, USA) with approximately ~90 nm of gold (Au). The thickness of the Au film was confirmed with an AFM measurement. The process of SLGr and FLBN transfer from the growth, Cu, onto flexible PET substrates was carried out via the wet transfer method. Briefly, the Gr/Cu or BN/Cu films were spin-coated with 950 molecular weights polymethyl methacrylate (PMMA) at 5000 rpm for 10 s to create a thin PMMA layer that can protect the grown films during the transfer. Next, samples coated with PMMA were suspended in a chemical solution and prepared with diluted ferric chloride in DI-water with a concentration of 1 M, in order to etch the Cu. Subsequently, the samples PMMA/SLGr or FLBN were cleaned from the Cu-residuals using an aqueous solution of hydrochloric iced diluted in DI-water (HCl/DI-water) with a volume ratio of (1:1). The PMMA/2D films were transferred to another petri-dish with DI-water for further cleaning before fishing and mounting them onto PET substrates. For the SLGr sample, the PMMA/SLGr was placed directly in the center of the PET substrate. Alternatively, to create the FLBN/SLGr heterostructure, the PMMA/FLBN sample was placed precisely on top of the already prepared SLGr/PET stacks. Lastly, the PMMA layer was removed from the surface of the heterostacks (SLGr/PET and FLBN/SLGr/PET) by soaking them in acetone overnight (10–12 h) for slow and gentle PMMA removal. Because of the presence of the PET substrate, rigorous thermal annealing of the heterostructure at a high temperature was not possible, hence some residual from the transfer process could be expected.

### 2.3. Mechanical Device (Jig) Characteristics

A custom-built “jig” apparatus was employed in our strain measurements. The mechanical jig utilizes a two-jaw construct, which allows for the movement of each jaw symmetrically in an opposing direction, so as to generate a bending strain, when the thumbwheel is rotated. A dovetail track is incorporated into the base of the apparatus. A matching shape milled into the bottom of the jaws constrains them to a single degree of freedom. By design, the jaws are made of bronze, while the track is made of stainless steel. This dissimilarity in materials prevents galling and wear over time. Flat plates screwed to the top of the jaws via miniature screws apply a clamping force to affix the ends of the substrate. As the jaws are brought together, a two-point bending strain is induced in the substrate. An opposed motion of the “jaws” is facilitated via rotating two threaded lead screws, which are directly geared together, resulting in counter-rotation. Rotation is induced in one lead screw via user input at a thumbwheel. Each jaw is only threaded for one of the two lead screws. Therefore, the jaws move in opposite directions when the thumbwheel is rotated. This allows for controlled movement of the apparatus with high precision using microscope stage-integrated controls. Measurement of the jaw displacement is facilitated through an integrated drop indicator with a resolution of one micrometer. The entire jig apparatus is fixed to an aluminum base plate, which mounts directly onto the upright microscope stage of the Raman spectroscopy system. It is worth noting that our universal custom-built jig can be utilized to characterize the mechanical properties of other 2D materials and heterostructures of different custom sizes prepared on a variety of flexible substrates. Moreover, it can also be used with other instruments, including various microscopy tools equipped with standard x-y-z-piezo-driven stages.

## 3. Results and Discussion

**Figure 1:**
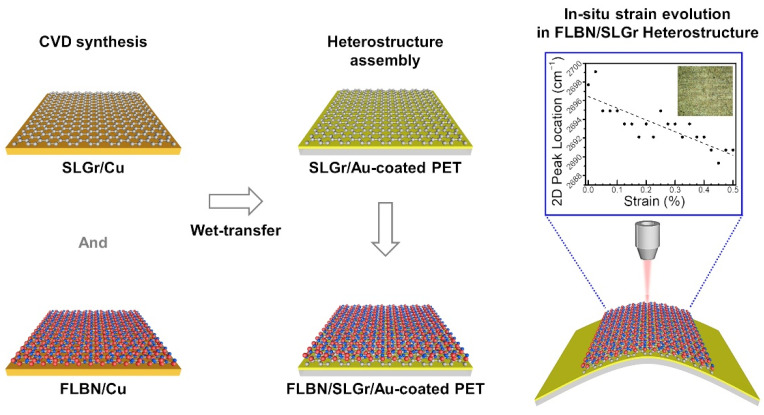
A schematic diagram illustrates the production process of FLBN/SLGr heterostructures on PET substrates. The left column shows the CVD growth of SLGr or FLBN on Cu substrates. The middle column shows the prepared Au-coated PET substrate followed by the FLBN/SLGr heterostructure assembly. The right column shows the schematic of the strained configuration of the FLBN/SLGr heterostructure on Au-coated PET substrates with in situ Raman spectroscopy (inset in the right column depicts a representative strain evolution measurement plot and optical image of the probed area).

In recent years, many studies have been devoted to understanding the specific mechanical properties of 2D materials, such as strain, strength, elasticity, and hardness, which are beneficial for specific applications, such as flexible electronic systems, optical sensors, or different types of wearable devices [8,45]. However, in most previously reported works, the mechanical properties of a single material, for example, individual Gr or BN thin films, have been investigated [10,14,15,16,19,28,29,46,47,48]. In contrast, only a few have focused on the mechanical testing of fabricated heterostructures [49]. This report has investigated one of the important mechanical characteristics (strain evolution) in the CVD-grown FLBN/SLGr heterostructures using a custom-built mechanical jig mounted to the stage of the Raman microscope to probe the strain effect in our samples in real time. Figure 1 schematically illustrates the essential steps in preparing FLBN/SLGr heterostructures on a flexible substrate for in situ measurements of strain evolution using Raman spectroscopy.

Briefly, a CVD system was first employed to prepare SLGr and FLBN on Cu foils in separate growth runs and then transferred onto the flexible substrate on top of each other. PET was Au-sputtered and utilized as flexible substrates. The Au film aids the enhancement of the resonant Raman scattering signal, in general, owing to the high-intensity charge transfers from the metal surface to the adsorbing species. Additional metal surface plasmonic effects also play a role [50]. The prepared SLGr was first transferred onto the Au-coated PET substrates. The heterostructure was assembled by placing FLBN films on top of the SLGr and mounted onto a mechanical device jig to study the strain evolution using Raman spectroscopy. Here, the inset in Figure 1 shows a snapshot of the representative measurement with an optical image of the probed area of the sample.

It is important to mention that Gr and BN behave differently when subjected to strain, while they are structurally similar (both having a hexagonal crystal lattice geometry). For instance, the mechanical strength of Gr is sensitive to the number of layers and diminishes as the number of layers increases. On the other hand, the strength of BN has not shown strong dependence on the number of layers. It was reported that the FLBN samples had exhibited similar strength to that of an SLBN sample [10]. In general, for BN, sliding energy is more significant than shear energy, preventing interlayers from sliding relative to one another [10,46], while between layers in Gr, shear energy is more significant than sliding energy. Therefore, it is essential to evaluate the strengths of not only individual 2D materials (Gr and BN), but also their few-layered heterostacks. This is because, during the application of strain to a multi-layer heterostack, the role of strain relaxation has not been studied well. An interesting interplay between the applied strain and measured strain could be when few-layered heterostacks are investigated for mechanical characterization.

**Figure 2:**
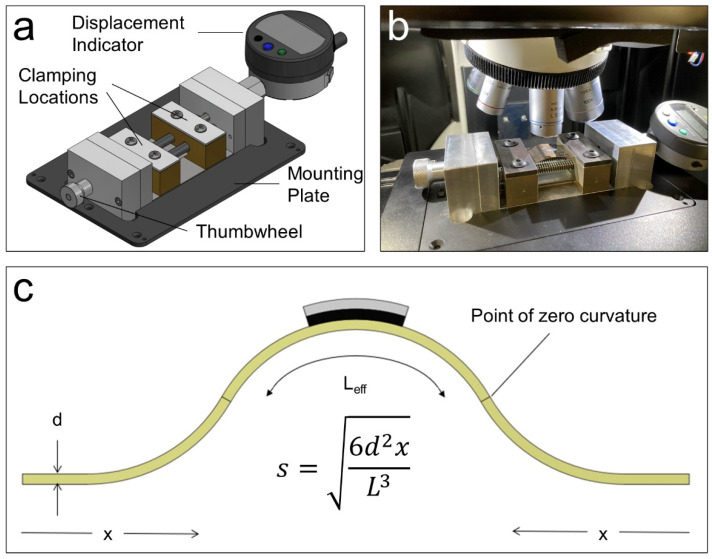
A custom-built jig used for inducing the strain effect on the prepared thin films. (**a**) A 3D model of the designed jig, including its main parts. (**b**) A photograph of the experimental setup, where a custom-built jig is mounted onto the motorized x-y-z stage of a commercial Raman spectroscopy instrument. Laser beam path lighted with the vertical axes of the microscope objective. (**c**) A schematic illustration of the prepared 2D heterostructure films on PET substrates and the relation between the strain *s* on the sample and the displacement *x* of the jig is also shown.

Accurate and consistent application of bending strain necessitated the design and construction of a custom mechanical device “jig” apparatus, as shown in Figure 2a. The details of the design of the jig are provided in the Section 2. It is most critical to maintain the focus area during the measurements, sample spot selection, and survey. Therefore, the device is mounted directly onto the Raman microscope’s linearized stage, as shown in Figure 2b. A two-point bending strain is induced in the substrate during measurement. From the geometric configuration of the substrate and the sample assuming uniform bending approximated as circular arcs, as shown in Figure 2c [34], the strain *s* imposed on the specimen through the interface between the substrate and 2D film, with thickness *d*, is $s=\frac{y}{r}=\frac{d/2}{r}=\frac{d}{2r}$, where *r* is the bending radius (in meters) and *y* is measured from the reference mid-plane of the substrate (in meters). This strain is, in turn, modulated by the displacement *x* of the jig (handle), with a micrometer resolution:

(1) $$x=L\left(1-\frac{d}{sL}\sin\frac{sL}{d}\right)$$

where *L* is the substrate width, which can also be approximated by the following:

(2) $$x\approx \frac{L^{3}s^{2}}{6d^{2}}$$

Thus, inverting the above equation, the strain *s* can be computed directly from measurable quantities via the following:

(3) $$s=\sqrt{\frac{6d^{2}x}{L^{3}}}$$

**Figure 3:**
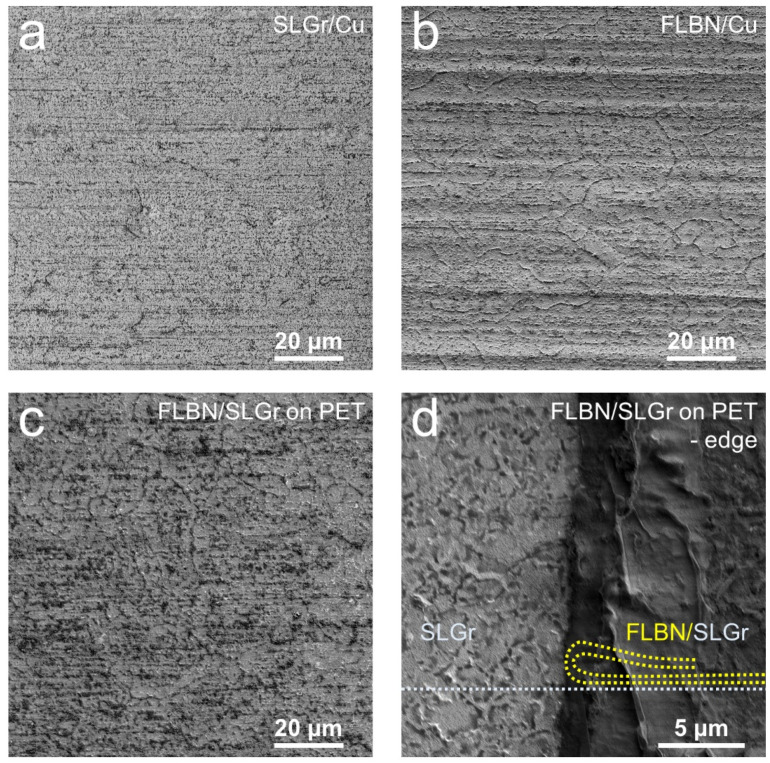
Surface characterization of as-grown SLGr, FLBN, and the prepared heterostructures. SEM images depict (**a**) SLGr and (**b**) FLBN grown on Cu foil. The SEM image in (**c**) shows large area surface morphology at the center part of the heterostructure stuck transferred onto PET substrate, while the image in (**d**) depicts a zoomed-in image of the edge area of the same FLBN/SLG heterostructure sample after applying the wet transfer procedure on Au-coated PET substrate.

The surface morphology of the CVD-grown sample was examined by scanning electron microscope (SEM; FEI Inspect F50, Lausanne, Switzerland). The SEM micrographs were obtained with a low acceleration voltage (1 keV) for better visualization of wrinkles and domain structures on surfaces of the SLG and FLBN samples. Figure 3a,b show SEM images of SLGr and FLBN as-grown on Cu foil. The SEM images of Figure 3c,d show the FLBN/SLGr heterostructure formed/assembled by wet transfer on Au-coated PET substrate after Cu etching (details of the transfer process can be found in the Section 2). We note that, in Figure 3c, we observed similar domain shapes or wrinkles on the surface of FLBN/SLGr/PET to those morphological outlines seen in Figure 3b for FLBN on Cu. Some dark spots visible in SEM images could be attributed to particles transferred from Cu foils, potential post-etching residual. On the other hand, at the edge of FLBN/SLGr/PET, a folded edge of FLBN can be seen (schematic dashed lines are shown for guiding purposes, and are overlayed as an inset in Figure 3d). Overall, the SEM characterization confirmed that SLGr and FLBN, as-grown and after transfer, are intact and have preserved the morphological characteristics of their surfaces.

**Figure 4:**
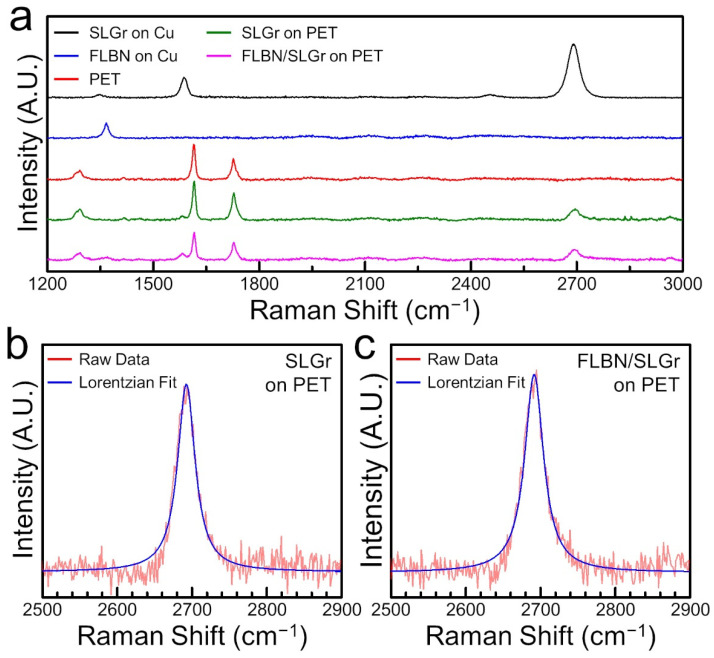
Raman spectroscopy characterization of the prepared SLGr and FLBN/SLGr heterostructures. (**a**) Raman spectral plot of a transferred SLGr on PET substrates (blue color), Raman plot of SLGr on Cu before transferring (black color), and that of PET substrate alone (red color). (**b**) Raman spectral plot of transferred FLBN/SLGr on PET substrates (magenta color), Raman plots of SLGr on Cu (black color) and FLBN on Cu (blue color) before transferring onto PET substrates, and PET substrate alone (red color). (**b**,**c**) Individual Raman spectral plots of *2D* bands of SLGr on PET and FLBN/SLGr on PET (blue color) (red lines in (**b**,**c**) represent the Lorentzian curve fits).

To characterize the Raman vibrational modes and track the strain evolution of the produced 2D materials (SLGr, FLBN, and FLBN/SLGr heterostructures), the 50× long working distance objective and 532 nm laser excitation were used in all measurements. First, the control characterizations were performed on the prepared thin films with no strain. Figure 4a shows the Raman spectral plots for SLGr on Cu alone (black), as-grown FLBN on Cu alone (blue), the Au-sputtered PET substrate (red), SLGr transferred onto Au-sputtered PET (green), and finally the FLBN/SLGr heterostructure on the Au-sputtered PET (magenta). The Raman analyses of the spectral plot containing as-grown SLGr on Cu have shown strong *G* (in-plane) and *2D* (second-order) vibrational modes of SLGr, represented by the peaks ~1585 cm^−1^ and ~2686 cm^−1^, respectively, and a small D-band at ~1340 cm^−1^, which is usually associated with the level of disorder or defects [1,2]. The Raman intensity ratio of *2D/G* bands of the synthesized SLGr is ~2.15, confirming that the prepared SLGr sample is a single layer. Raman spectral plots for as-grown FLBN show a sharp peak, attributed to the first-order in-plane mode located at ~1360 cm^−1^, which is a characteristic of h-BN [5,51]. Likewise, for SLGr/PET samples in Figure 4a, the materials’ (SLGr, PET) characteristic Raman peaks were identified individually to ensure the preservation of the materials’ quality in each subsequent step prior to assembling the heterostructure. After the last step of transferring the FLBN film on top of the SLGr/PET samples, the Raman spectral plot was collected. We found that the main vibrational modes represented by the Raman peaks at ~1360 cm^−1^, ~1614 cm^−1^, and ~2686 cm^−1^ confirm the presence of FLBN, PET, and SLGr in the prepared heterostructure samples, respectively. The peaks represented in the spectral plot for Au-sputtered PET (red) at ~1614 cm^−1^ and ~1726 cm^−1^ were indicative of the PET vibrational modes [4] and used as a reference. It can be seen that most spectral plots in Figure 4a have shown a very shallow oscillatory background in the range of 1900 cm^−1^–2900 cm^−1^, which was attributed to internal environmental factors of the Raman spectrometer, although it is possible that external factors such as mechanical vibrations/instabilities could also play a role. We also note that our Raman measurements were conducted with multiple spectral accumulations to enhance the signal-to-noise ratio of the measured Raman signal. Spectra were collected and averaged from each characterized spot and for each sample. Here, special attention was paid to ensure that, during the experiments, laser power was minimal, so that no heating would occur. With our configuration of the instrument, long working distance objectives, and so on, power was estimated to be around ~1–2 mW [52], hence not significant (see details in the Section 2).

To evaluate the amount of disorder, we conducted Raman quantitative analyses of the intensity ratio between the *G-*band and *D-*band, (I_G_/I_D_), and the so-called ‘quality factor’ (QF) was also evaluated. It was found that the ratios (I_G_/I_D_) for SLGr/Cu, SLGr/PET, and FLBN/SLGr had QF values of 6.33, 4.24, and 3.71, respectively. This QF factor is often used to evaluate the averaged qualitative and quantitative measures of the disorder in the materials. Indeed, Raman characterization of the *D-*band indicated that as-grown SLGr on Cu substrate had the highest QF, which subsequently decreased with each transfer step. While visual inspection via optical microscopy and SEM did not show notable changes on the surface of 2D materials, we assume some physisorbed or chemisorbed residuals from the wet transfer process have played a role in the slightly diminished QF values, which imply to the increased disorder and/or defects at the interfaces or on the surface of the assembled heterostructure. It is worth noting that the location of the PET band peak at ~1614 cm^−1^ has a high intensity and interfered with the *G*-band peak of the SLGr at ~1600 cm^−1^ [3]. Hence, we focused our studies on the *2D* band of SLGr on PET and FLBN/SLGr on PET heterostructure samples and tracked the strain evolution using shifts measured in the *2D* band in the subsequent experiments described below.

For accuracy and consistency in evaluating the strain evolution in SLGr and FLBN/SLGr heterostructures, accurate determination of the *2D* band peak locations is critical. The background noise inherent in Raman spectroscopy sometimes resulted in the obfuscation of the precise location of the peaks. For this reason, a Lorentzian fit was applied to the spectrum between 2500 cm^−1^ and 2900 cm^−1^ to aid in obtaining the exact location of the peak. Figure 4b,c show the Raman *2D* peak of SLGr with a fitted Lorentzian curve overlaid. The Raman peaks of SLGr and other materials have been shown to follow a Lorentzian distribution [38]. The full widths at half maximum (FWHM) for these Lorentzian fitted plots for the SLGr and the FLBN/SLGr heterostructure samples were approximately 29 cm^−1^ and 32 cm^−1^, respectively, indicating a good quality/crystallinity of the SLGr, which is consistent with other reported studies [53]. Once the initial characterization is completed, strain can then be applied to the substrate to determine the effect of stress on SLGr and heterostructure.

**Figure 5:**
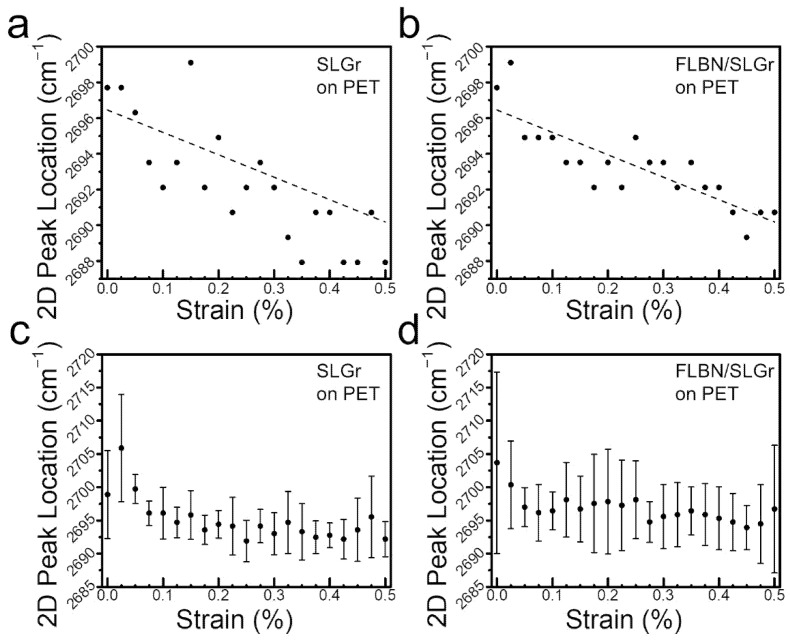
The evolution of the *2D* bands of the prepared SLGr alone and within FLBN/ SLGr heterostructures on PET at various strains. (**a**) A representative plot of an individual SLGr 2D peak’s evolution from 0% to 0.5% strain. (**b**) A representative plot of the 2D peak’s evolution within the SLGr/FLBN heterostructure from 0% to 0.5% strain. (**c**,**d**) The average 2D peak location vs. strain for the entire SLGr and FLBN/SLGr heterostructure, respectively. The error bars correspond to one standard deviation away from the mean.

To achieve this, a custom-built jig was used to induce strain in the SLGr and the FLBN/SLGr heterostructure samples. Multiple measurements were taken at various intervals of the spectrum over the entire range of strain. The sizing of our mechanical device/jig allowed for a strain regime from 0 to 0.5% strain, with a resolution of 0.025% strain, thus giving 21 unique strain measurements. Figure 5a,b show the change in the peak position of the Gr *2D* band in the prepared SLGr and FLBN/SLGr heterostructures as a function of the applied strain in the range of 0–0.5%. It can be noticed that the *2D* peak in the SLGr alone shifted linearly by −17.9 cm^−1^/% strain, as shown in Figure 5a. However, the shift value in the case of SLGr/FLBN heterostructures was found to be −12.5 cm^−1^/% strain, as shown in Figure 5b. Thus, the *2D* band of the SLGr sheet alone shows more substantial strain-dependent red shifts (towards lower frequencies in cm^−1^) as compared with the FLBN/SLGr heterostructure under the same strain. This implies that, in SLGr, carbon–carbon bond elongation due to applied strain is more prominent in SLGr on PET than in the heterostructure FLBN/SLGr on PET substrate. Therefore, we speculate that the FLBN thin film serves as a cap layer/reducer of the applied strain, which is manifested in Raman spectral plots measured from SLGr underneath it. These results are compatible with the recently reported work by Yang et al. [45], which implied that the few-layered h-BN of 2 to 6 layers has bending stiffness following the power function of thickness, and can be stronger than MLGr by an order of magnitude; owing to its intrinsic structure, the ionic B-N bonding [19,48] possesses high fracture toughness with stable crack propagation [46].

Figure 5c,d show averaged trends across five different spots studied within the sample. Here, the error bars indicate one standard deviation of variance between multiple spots over multiple measurements. The data illustrated by these two graphs combined five individual strain regimes of SLGr and FLBN/SLGr on PET, respectively. This larger dataset size includes several different locations on the sample. The best-fit slope for the averaged strain regimes of the SLGr alone was ~19.88 cm^−1^/% strain, while the best-fit slope for the averaged strain regimes of the FLBN/SLGr heterostructure was ~15.27 cm^−1^/% strain. The relatively high variance in the first two data points is notable in Figure 5c,d. This is because the initial step from 0% strain to 0.025% strain requires a 1-micrometer movement in the jaws, and the resolution of the drop-indicator is 1 micrometer. This error is fully mitigated in the rest of the experiment because the necessary movement of the jaws increases for each successive 0.025% strain interval. This effect results from the relationship between the jaw displacement (*x*) and the strain applied to the sample, which can be explained with Equation (2) above, where the required displacement (*x*) is proportional to the square of the desired strain. The subsequent outcome of this constraint is that the first strain interval requires a 1-micrometer adjustment of the jaws, while the final strain interval, 0.475% to 0.500% strain, requires a 44-micrometer adjustment of the jaws. Thus, the accuracy of the desired displacement is 44 times greater on the last step of the strain regime than on the first. For this reason, there will be increased variance at the lower strains and lower variance as the regime progresses towards the higher strain.

Our results consistently show that the downward shift value of the *2D* band/peak for SLGr on the PET sample is larger than that of the heterostructure FLBN/SLGr on PET. These results of a consistent downward shift in the location of the SLGr *2D* band/peak under progressive strain are in general agreement with other reported studies for SLGr [34,54]. In our work, furthermore, the FLBN/SLGr heterostructure similarly shows this trend, though slightly less pronounced. This is an intriguing result. The reduction in the amount of the down-shift in the *2D* band/peak for the heterostructure sample as compared with the SLGr implies that the presence of FLBN in the heterostructure effectively reduces the strain transferred to the SLGr. The consistency of these measurements over multiple loading strain cycles shows the resiliency of the FLBN/SLGr heterostructure to stress as compared with the SLGr material. This is observed from a 30% lesser Raman shift in the *2D* band/peak, which could be correlated to a reduced strain within the sample. We, therefore, assume that the strength/durability is enhanced in the FLBN/SLGr heterostructure as compared with SLGr.

## 4. Conclusions

Investigations focused on understanding the mechanical properties of 2D materials and their heterostructure have attracted tremendous research interest owing to their unique structural characteristics. Two-dimensional materials are strain-sensitive, their lattice structure can be tuned, and thus their electronic and optoelectronic properties can be engineered. Using the Raman spectroscopy technique, we have successfully measured the in situ strain evolution of FLBN/SLGr heterostructures in this study. Here, the 2D materials are fabricated as vertical heterostructures on top of the flexible (PET) substrates. Using the chemical vapor deposition method for the synthesis of SLGr and FLBN enabled the production of uniform thin films, which significantly improved the fidelity of the measurements and overall throughput, unlike other reported cases where exfoliated flakes were used to prepare individual 2D layers or heterostructure assemblies. The interactions of the fabricated heterostructures have shown a substantial impact on the band structures, which is corroborated by the amount of shift in the in-plane vibrational modes in the range of 0–0.5% strain. The obtained results show that the *2D* band in SLGr alone shows 30% more substantial strain-dependent frequency shifts than that of SLGr assembled in the FLBN/SLGr heterostructure under the same strain. These observations imply that, in the heterostructure, the Gr layer can “effectively” experience reduced applied strain. This could influence energy dissipation through each layer in the weakly coupled few-layered vdW materials.

The strain measurements performed with the in situ Raman spectroscopy on the large area (up to 5 × 5 mm) heterostructures prepared on flexible PET substrates have provided more reliable and reproducible statistical outcomes. Measuring a large area of synthesized CVD-thin films on flexible substrates using a non-destructive method could help in estimating/predicting the effect of strain in flexible and stretchable devices, where the FLBN/SLGr combination is often used [38,55,56,57,58]. Moreover, this technique and its relatively flexible assembly can be more applicable when studying larger samples as compared with suspended smaller films/flakes used in AFM-assisted mechanical testing. While etching and wet transfer protocols were used for assembling heterostructures on PET substrates, it could be possible to consider alternative preparation techniques to minimize potential contaminants often associated with wet transfer methods. Our custom-built mechanical device-jig embedded onto the stage holder of the microscope allows for precise (sub-micrometer) tracking of probed area/spot at all amounts of strain. This feature ensures that each measurement is performed on the exact location of the film throughout the experiment and prevents inconsistencies from potential structural differences that could be notable between adjacent domains in the SLGr. We also note that our universal mechanical device-jig can also be utilized to study the strain effect in various 2D materials and heterostructures prepared on flexible substrates using in situ Raman spectroscopy.

In addition to the ordinary mechanical properties, 2D materials possess many outstanding physical properties, such as electrical, magnetic, thermal, and optical properties, to name a few [38]. By further development of fabrication techniques and through assembling different 2D materials into heterostructures, these physical properties can be utilized in specific applications for novel nanoelectronics, optoelectronics, spintronics, and energy applications, as well as for medical or biomedical devices [59]. A better understanding of the mechanical properties of strained, layered 2D materials, especially heterostructures, can improve the design and fabrication of future flexible and stretchable devices and multidimensional platforms for various targeted needs. More computational studies focused on heterostructures is critical, especially those that consider the substrate effects, interlayer interaction, as well as the roles of defects/disorders [60,61]. For a variety of practical applications, 2D materials and heterostructures prepared via CVD-growth are critical. Some studies showed that defects such as voids and grain boundaries formed during synthesis may increase or decrease the elastic stiffness and strength of Gr, depending on the density of the defects and the configuration [19,37,39,49,62,63]. While detailed studies of defect contents in our materials are not correlated directly to our measurement of strain evolution in FLBN/SLGr heterostructures, Raman analyses provided an averaged evaluation of our heterostructure quality and the level of disorder. We believe it would be useful in the future to design a device/system that would allow for such an approach where systematic analyses of strain evolution in multi-layered heterostructure can be correlated to the density of defects. Nevertheless, this work is a first demonstration of the possibility to study the FLBN/SLGr heterostructure, or other vdW 2D materials in the future, with the Raman approach more rapidly and on a larger scale.

## Data Availability

The data that support the findings of this study are available from the corresponding author upon reasonable request.

## References

[1] Khan K., Tareen A.K., Aslam M., Wang R., Zhang Y., Mahmood A., Ouyang Z., Zhang H., Guo Z. (2020). Recent developments in emerging two-dimensional materials and their applications. J. Mater. Chem. C.

[2] Glavin N.R., Rao R., Varshney V., Bianco E., Apte A., Roy A., Ringe E., Ajayan P.M. (2020). Emerging Applications of Elemental 2D Materials. Adv. Mater..

[3] Alameri D., Nasr J.R., Karbach D., Liu Y., Divan R., Das S., Kuljanishvili I. (2019). Mask-free patterning and selective CVD-growth of 2D-TMDCs semiconductors. Semicond. Sci. Technol..

[4] Alameri D., Ocola L.E., Kuljanishvili I. (2018). Mask-free fabrication and chemical vapor deposition synthesis of ultrathin zinc oxide microribbons on Si/SiO2 and 2D substrates. J. Vac. Sci. Technol. A.

[5] Liang S.-J., Cheng B., Cui X., Miao F. (2020). Van der Waals Heterostructures for High-Performance Device Applications: Challenges and Opportunities. Adv. Mater..

[6] Choi W., Choudhary N., Han G.H., Park J., Akinwande D., Lee Y.H. (2017). Recent development of two-dimensional transition metal dichalcogenides and their applications. Mater. Today.

[7] Ye M., Zhang D., Yap Y.K. (2017). Recent Advances in Electronic and Optoelectronic Devices Based on Two-Dimensional Transition Metal Dichalcogenides. Electronics.

[8] Pang Y., Yang Z., Yang Y., Ren T.-L. (2020). Wearable Electronics Based on 2D Materials for Human Physiological Information Detection. Small.

[9] Wang J., Ma F., Liang W., Wang R., Sun M. (2017). Optical, photonic and optoelectronic properties of graphene, h-BN and their hybrid materials. Nanophotonics.

[10] Falin A., Cai Q., Santos E.J.G., Scullion D., Qian D., Zhang R., Yang Z., Huang S., Watanabe K., Taniguchi T. (2017). Mechanical properties of atomically thin boron nitride and the role of interlayer interactions. Nat. Commun..

[11] Wang J., Ma F., Liang W., Sun M. (2017). Electrical properties and applications of graphene, hexagonal boron nitride (h-BN), and graphene/h-BN heterostructures. Mater. Today Phys..

[12] De Sanctis A., Mehew J.D., Alkhalifa S., Withers F., Craciun M.F., Russo S. (2018). Strain-Engineering of Twist-Angle in Graphene/hBN Superlattice Devices. Nano Lett..

[13] Dresselhaus M.S., Dresselhaus G., Saito R., Jorio A. (2005). Raman spectroscopy of carbon nanotubes. Phys. Rep..

[14] Liu L., Liu Y., Li X., Liu N., Cui B., Huang X. (2019). Bending Effects in Graphene: Implication for Flexible Transparent Electronics. Phys. Status Solidi (b).

[15] Sahalianov I.Y., Radchenko T.M., Tatarenko V.A., Cuniberti G., Prylutskyy Y.I. (2019). Straintronics in graphene: Extra large electronic band gap induced by tensile and shear strains. J. Appl. Phys..

[16] Lee C., Wei X., Kysar J.W., Hone J. (2008). Measurement of the Elastic Properties and Intrinsic Strength of Monolayer Graphene. Science.

[17] Bilal M., Xu W., Wang C., Wen H., Zhao X., Song D., Ding L. (2020). Optoelectronic Properties of Monolayer Hexagonal Boron Nitride on Different Substrates Measured by Terahertz Time-Domain Spectroscopy. Nanomaterials.

[18] Kostoglou N., Polychronopoulou K., Rebholz C. (2015). Thermal and chemical stability of hexagonal boron nitride (h-BN) nanoplatelets. Vacuum.

[19] Han Y., Feng S., Cao K., Wang Y., Gao L., Xu Z., Lu Y. (2020). Large Elastic Deformation and Defect Tolerance of Hexagonal Boron Nitride Monolayers. Cell Rep. Phys. Sci..

[20] Song X., Gao T., Nie Y., Zhuang J., Sun J., Ma D., Shi J., Lin Y., Ding F., Zhang Y. (2016). Seed-Assisted Growth of Single-Crystalline Patterned Graphene Domains on Hexagonal Boron Nitride by Chemical Vapor Deposition. Nano Lett..

[21] Aggoune W., Cocchi C., Nabok D., Rezouali K., Akli Belkhir M., Draxl C. (2017). Enhanced Light–Matter Interaction in Graphene/h-BN van der Waals Heterostructures. J. Phys. Chem. Lett..

[22] Maggini L., Ferreira R.R. (2021). 2D material hybrid heterostructures: Achievements and challenges towards high throughput fabrication. J. Mater. Chem. C.

[23] Xu H.Y., Akbari M.K., Zhuiykov S. (2021). 2D Semiconductor Nanomaterials and Heterostructures: Controlled Synthesis and Functional Applications. Nanoscale Res. Lett..

[24] Zhang S.Q., Liu J.L., Kirchner M.M., Wang H., Ren Y.L., Lei W. (2021). Two-dimensional heterostructures and their device applications: Progress, challenges and opportunities-review. J. Phys. D Appl. Phys..

[25] Pantano M.F., Kuljanishvili I. (2020). Advances in mechanical characterization of 1D and 2D nanomaterials: Progress and prospects. Nano Express.

[26] Suk J.W., Piner R.D., An J., Ruoff R.S. (2010). Mechanical Properties of Monolayer Graphene Oxide. ACS Nano.

[27] Wang C., Frogley M.D., Cinque G., Liu L.-Q., Barber A.H. (2013). Deformation and failure mechanisms in graphene oxide paper using in situ nanomechanical tensile testing. Carbon.

[28] Neumann C., Reichardt S., Venezuela P., Drögeler M., Banszerus L., Schmitz M., Watanabe K., Taniguchi T., Mauri F., Beschoten B. (2015). Raman spectroscopy as probe of nanometre-scale strain variations in graphene. Nat. Commun..

[29] del Corro E., Taravillo M., Baonza V.G. (2012). Nonlinear strain effects in double-resonance Raman bands of graphite, graphene, and related materials. Phys. Rev. B.

[30] Li Z., Kinloch I.A., Young R.J. (2016). The role of interlayer adhesion in graphene oxide upon its reinforcement of nanocomposites. Philos. Trans. R. Soc. A Math. Phys. Eng. Sci..

[31] Androulidakis C., Koukaras E.N., Parthenios J., Kalosakas G., Papagelis K., Galiotis C. (2015). Graphene flakes under controlled biaxial deformation. Sci. Rep..

[32] Ni Z.H., Yu T., Lu Y.H., Wang Y.Y., Feng Y.P., Shen Z.X. (2008). Uniaxial Strain on Graphene: Raman Spectroscopy Study and Band-Gap Opening. ACS Nano.

[33] Mohiuddin T.M.G., Lombardo A., Nair R.R., Bonetti A., Savini G., Jalil R., Bonini N., Basko D.M., Galiotis C., Marzari N. (2009). Uniaxial strain in graphene by Raman spectroscopy: $G$ peak splitting, Gr\"uneisen parameters, and sample orientation. Phys. Rev. B.

[34] Chhikara M., Gaponenko I., Paruch P., Kuzmenko A.B. (2017). Effect of uniaxial strain on the optical Drude scattering in graphene. 2D Mater..

[35] Malard L.M., Pimenta M.A., Dresselhaus G., Dresselhaus M.S. (2009). Raman spectroscopy in graphene. Phys. Rep..

[36] Cong X., Liu X.L., Lin M.L., Tan P.H. (2020). Application of Raman spectroscopy to probe fundamental properties of two-dimensional materials. Npj 2D Mater. Appl..

[37] Cancado L.G., Jorio A., Ferreira E.H.M., Stavale F., Achete C.A., Capaz R.B., Moutinho M.V.O., Lombardo A., Kulmala T.S., Ferrari A.C. (2011). Quantifying Defects in Graphene via Raman Spectroscopy at Different Excitation Energies. Nano Lett..

[38] Wang J., Ma F., Sun M. (2017). Graphene, hexagonal boron nitride, and their heterostructures: Properties and applications. RSC Adv..

[39] Androulidakis C., Zhang K., Robertson M., Tawfick S. (2018). Tailoring the mechanical properties of 2D materials and heterostructures. 2D Mater..

[40] Dean C.R., Young A.F., Meric I., Lee C., Wang L., Sorgenfrei S., Watanabe K., Taniguchi T., Kim P., Shepard K.L. (2010). Boron nitride substrates for high-quality graphene electronics. Nat. Nanotechnol..

[41] Pizzocchero F., Gammelgaard L., Jessen B.S., Caridad J.M., Wang L., Hone J., Bøggild P., Booth T.J. (2016). The hot pick-up technique for batch assembly of van der Waals heterostructures. Nat. Commun..

[42] Kim Y., Herlinger P., Taniguchi T., Watanabe K., Smet J.H. (2019). Reliable Postprocessing Improvement of van der Waals Heterostructures. ACS Nano.

[43] Du J., Yu H., Liu B., Hong M., Liao Q., Zhang Z., Zhang Y. (2021). Strain Engineering in 2D Material-Based Flexible Optoelectronics. Small Methods.

[44] Rouhi S., Pourmirzaagha H., Farzin A. (2019). Predicting the mechanical properties of multi-layered silicene by molecular dynamics simulations. Mater. Res. Express.

[45] Yang Y., Song Z., Lu G., Zhang Q., Zhang B., Ni B., Wang C., Li X., Gu L., Xie X. (2021). Intrinsic toughening and stable crack propagation in hexagonal boron nitride. Nature.

[46] Qu W., Bagchi S., Chen X., Chew H.B., Ke C. (2019). Bending and interlayer shear moduli of ultrathin boron nitride nanosheet. J. Phys. D Appl. Phys..

[47] Li C., Bando Y., Zhi C., Huang Y., Golberg D. (2009). Thickness-dependent bending modulus of hexagonal boron nitride nanosheets. Nanotechnology.

[48] Tsuneda T., Iwasa T., Taketsugu T. (2019). Roles of silver nanoclusters in surface-enhanced Raman spectroscopy. J. Chem. Phys..

[49] Liu K., Wu J. (2016). Mechanical properties of two-dimensional materials and heterostructures. J. Mater. Res..

[50] Reich S., Ferrari A.C., Arenal R., Loiseau A., Bello I., Robertson J. (2005). Resonant Raman scattering in cubic and hexagonal boron nitride. Phys. Rev. B.

[51] Dresselhaus M.S., Jorio A., Hofmann M., Dresselhaus G., Saito R. (2010). Perspectives on Carbon Nanotubes and Graphene Raman Spectroscopy. Nano Lett..

[52] Zhang Y.Y., Son H., Zhang J., Kong J., Liu Z.F. (2007). Laser-heating effect on Raman spectra of individual suspended single-walled carbon nanotubes. J. Phys. Chem. C.

[53] Ferrari A.C., Meyer J.C., Scardaci V., Casiraghi C., Lazzeri M., Mauri F., Piscanec S., Jiang D., Novoselov K.S., Roth S. (2006). Raman Spectrum of Graphene and Graphene Layers. Phys. Rev. Lett..

[54] Tsoukleri G., Parthenios J., Galiotis C., Papagelis K. (2015). Embedded trilayer graphene flakes under tensile and compressive loading. 2D Mater..

[55] Yankowitz M., Ma Q., Jarillo-Herrero P., LeRoy B.J. (2019). van der Waals heterostructures combining graphene and hexagonal boron nitride. Nat. Rev. Phys..

[56] Li Q.C., Liu M.X., Zhang Y.F., Liu Z.F. (2016). Hexagonal Boron Nitride-Graphene Heterostructures: Synthesis and Interfacial Properties. Small.

[57] Novoselov K.S., Mishchenko A., Carvalho A., Neto A.H.C. (2016). 2D materials and van der Waals heterostructures. Science.

[58] Geim A.K., Grigorieva I.V. (2013). Van der Waals heterostructures. Nature.

[59] Chakraborty S.K., Kundu B., Nayak B., Dash S.P., Sahoo P.K. (2022). Challenges and opportunities in 2D heterostructures for electronic and optoelectronic devices. Iscience.

[60] Zhong T., Li J.B., Zhang K.W. (2019). A molecular dynamics study of Young’s modulus of multilayer graphene. J. Appl. Phys..

[61] Liu Y., Pan Y.C., Yin D.Q., Song S.F., Lin L.Y., Zhang M.X., Qi X.L., Yao J.Y. (2021). Mechanical properties and thickness-determined fracture mode of hexagonal boron nitride nanosheets under nanoindentation simulations. Comput. Mater. Sci..

[62] Wei Y.J., Wu J.T., Yin H.Q., Shi X.H., Yang R.G., Dresselhaus M. (2012). The nature of strength enhancement and weakening by pentagon-heptagon defects in graphene. Nat. Mater..

[63] Lopez-Polin G., Gomez-Navarro C., Parente V., Guinea F., Katsnelson M.I., Perez-Murano F., Gomez-Herrero J. (2015). Increasing the elastic modulus of graphene by controlled defect creation. Nat. Phys..

